# Cost Savings of Using Fluorescein Strips in Laser Laryngoscopy

**DOI:** 10.7759/cureus.102985

**Published:** 2026-02-04

**Authors:** Michael P Gebhard, Heather McDougall, Neil N Chheda

**Affiliations:** 1 Otolaryngology - Head and Neck Surgery, University of Florida College of Medicine, Gainesville, USA

**Keywords:** cost, fluorescein, laryngology, laser, methylene blue

## Abstract

This technical report describes a simple, yet novel, approach to endotracheal cuff inflation in laryngological laser surgery that may result in significant cost savings for hospitals and patients. Pricing information was obtained from our tertiary care hospital’s operating room pharmacy as of March 3, 2025. A common safety practice when using carbon dioxide (CO2) or potassium titanyl phosphate (KTP) lasers is to fill the endotracheal cuff balloon with normal saline and methylene blue. The cost of the smallest vial of methylene blue is $150.00, and the entire bottle must be discarded after each case. By using fluorescein strips, the cost of this safety measure can be reduced to $0.16. The option of using ophthalmic fluorescein strips is 0.11% of the cost of using methylene blue and effectively brings the cost close to zero. Therefore, by using fluorescein strips, laser laryngeal surgeries can realize a 99.89% savings in the balloon cuff component of the procedure. We propose using fluorescein dye in place of methylene blue for endotracheal cuffs in laser laryngeal surgeries. This change is easy to implement and may result in immediate, significant cost savings for both patients and hospitals.

## Introduction

Laser surgery is used in a variety of laryngological procedures for the advantages of providing improved accuracy of incisions, better hemostasis, and the ability to vaporize tissue [1]. Although this technology has definite benefits, additional costs are incurred due to increased operating room time, additional staffing requirements, and safety precautions. The subject of our discussion is focused on reducing the cost of one specific safety precaution, laser cuff inflation.

## Technical report

Common safety practice with the use of CO2 or KTP lasers is to fill the endotracheal cuff balloon with normal saline and methylene blue [2,3]. This serves as an indicator of cuff perforation from the laser. The prices of materials were obtained from the University of Florida’s operating room pharmacy starting in 2019 and, most recently, on March 3, 2025.

**Figure 1:**
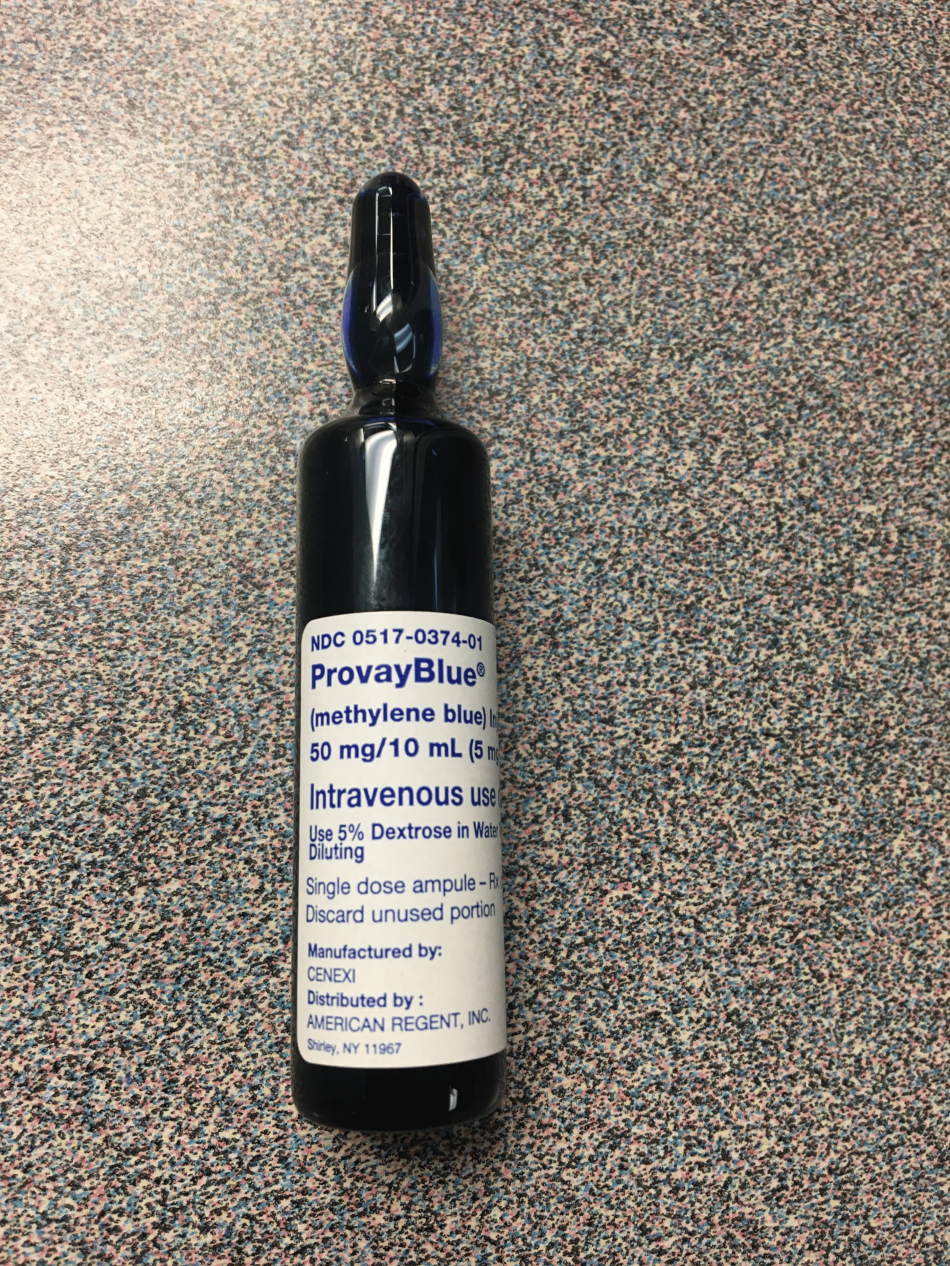
10 milliliter (mL) vial of 1% methylene blue

The cost of a 10 milliliter (mL) vial of 1.0% methylene blue (ProvayBlue; manufactured by CENEX and distributed by American Regent, Inc.) was $178.98 in July 2019. Despite only needing a few drops of methylene blue for this protective measure, an entire vial must be opened and subsequently discarded after each case. This added expense can be drastically mitigated with our proposed solution (Figure 1).

For the past ten years, the laryngologist at the University of Florida has been using fluorescein in place of methylene blue to fill the cuff balloon for laser laryngeal surgery. Like methylene blue, fluorescein has no toxicity to the respiratory mucosa [4-6] and can be a definitive indicator of cuff perforation. The main difference between fluorescein and methylene blue is the cost. The cost of a 5 mL vial of 10% fluorescein was $22.78 in 2019, which would have saved 87.3% of the cost of using methylene blue. Using a vial of fluorescein as an alternative to methylene blue provides immediate cost savings to the patient and to the hospital.

**Figure 2:**
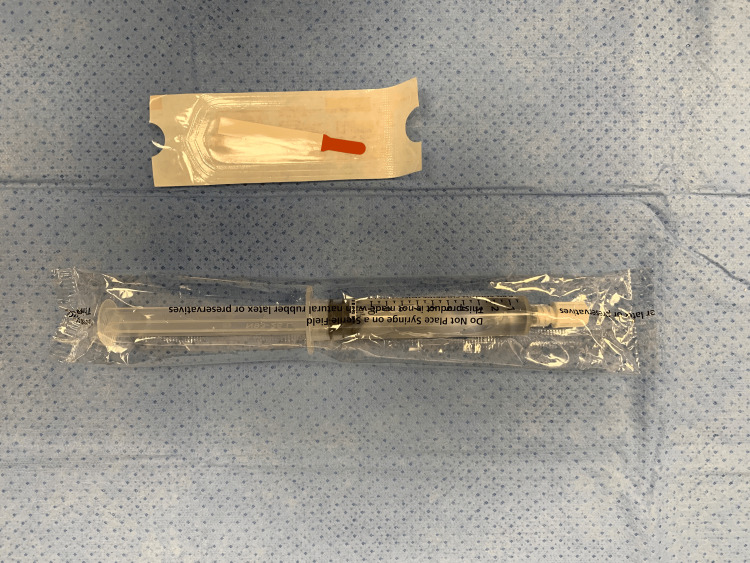
Ophthalmic fluorescein strip with 10 mL saline flush.

**Figure 3:**
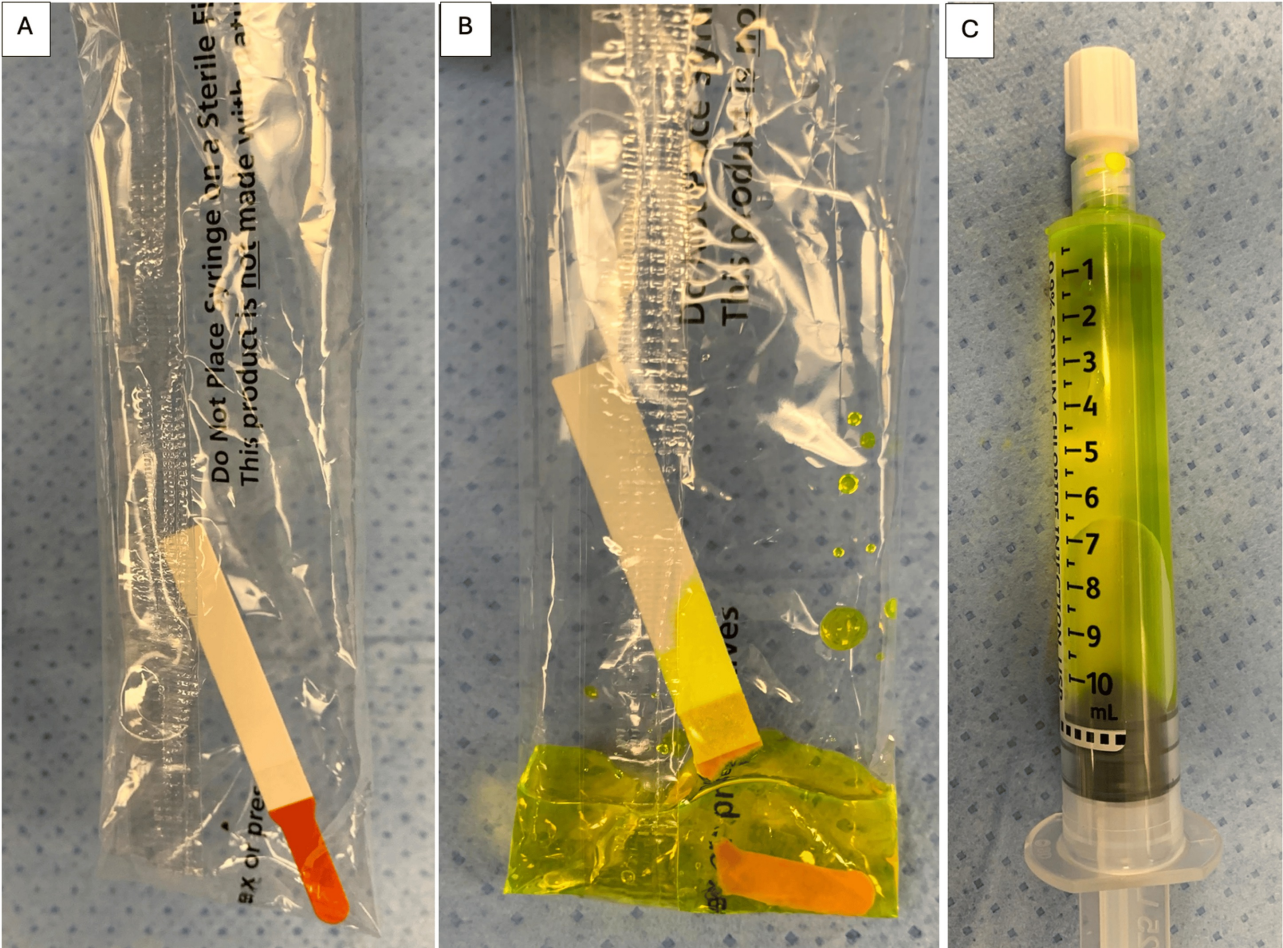
A. The fluorescein strip is placed in the saline flush packaging. B. Saline is added into the flush packaging. C. The new fluorescein solution is drawn up into flush syringe.

A further reduction of expenses can be achieved if ophthalmic fluorescein strips are used. Each sterile strip contains 1 mg of fluorescein sodium. The dye solution is easily prepared in the operating room by placing one fluorescein strip in a saline flush wrapper and adding several milliliters of saline (Figure 2). The dye is created instantly and then drawn back into the 10 mL saline flush syringe (Figure 3). The resultant solution concentration is about 0.1 mg/mL of fluorescein. In some scenarios, multiple flush syringes are prepared with the same fluorescein strip, so the actual concentration is sometimes lower.

The cost of a box of Bio-Glo 100-count ophthalmic fluorescein strips distributed by Hub Pharmaceuticals LLC was $15.97 in 2019, which was 91.1% less expensive than the cost of a single vial of methylene blue. The box of fluorescein strips does not expire for 36 months, and each strip comes individually packaged. This allows for greater efficiency and cost savings, as only a single strip needs to be used for each case and the box of fluorescein strips can be stored in the OR for future cases, unlike methylene blue. If a single box of fluorescein strips is used for 100 cases, the average cost per procedure is $0.16, compared to the $178.98 vial of methylene blue. The option of using an ophthalmic fluorescein strip was 99.91% less expensive than the cost of using methylene blue in 2019. By using fluorescein strips, laser laryngeal surgeries could realize a 99.91% savings on the balloon cuff component of the procedure, virtually zero.

As of March 2025, the price of a 10 mL vial of 1.0% methylene blue is $150.00. The price of a 5 mL vial of 10% fluorescein is $48.00, and the price of a box of 100-count ophthalmic fluorescein strips is $15.25 (Table 1). Comparing the cost of these three products over the past six years, the prices of the vial of methylene blue and the fluorescein strips both decreased by 16.19% and 4.51%, respectively, while the price of a fluorescein vial increased by 110.71%. Despite these changes, the cost discrepancy between one vial of methylene blue and a box of fluorescein strips remains similar. At the $15.25 price for a 100-count box of ophthalmic fluorescein strips, each strip costs approximately $0.16, which is 99.89% less expensive than the cost of a vial of methylene blue. Therefore, despite these price changes, utilizing ophthalmic fluorescein strips for the endotracheal balloon produces significant cost savings. These savings were seen in 2019 when the analysis was first performed and continue to the present time.

**Table 1:** Cost savings of using fluorescein compared with methylene blue.

Method	2019 cost	2025 cost	Price change from 2019 to 2025 (%)	Cost savings*	% Cost savings*
Methylene blue	$178.98	$150.00	-16.19%	-	-
5 mL vial of fluorescein	$22.78	$48.00	1.1071	$102.00	68%
100-count box of fluorescein strips	$15.97	$15.25	-4.51%	-	-
1 fluorescein strip	$0.16	$0.16	0%	$149.84	99.89%

## Discussion

In the current atmosphere of increasing health care costs in the United States, physicians and hospital administrators need to be proactive in adapting innovative ideas that reduce cost. The best innovations provide equal or improved quality of health care services while simultaneously reducing costs. We propose using fluorescein strips in place of methylene blue for endotracheal cuff inflation in laser laryngeal surgeries. This intervention effectively eliminates the cost of the laser cuff inflation component of the procedure and does not reduce the quality of care in any significant way. Adhering to this simple suggestion over time will lead to substantial savings for hospitals and patients.

Methylene blue has been widely used in medical practice, including identifying intestinal, enterovesical, and bronchopleural fistulas by providing direct visual confirmation of leaks when administered through different anatomical spaces [7,8]. Methylene blue has also been one of the default dyes in airway management, particularly for detecting endotracheal cuff leaks [2,3]. Traditionally, it is added to the cuff inflation medium, and if the cuff is compromised, the dye can be observed in tracheal secretions or detected during suctioning [2,3].

Fluorescein dye offers the same diagnostic capability for detecting endotracheal cuff leaks. Fluorescein is already widely used in ophthalmology, vascular studies, and airway procedures [5-6,9-10]. During cardiac surgery to repair multiple ventricular septal defects, it has been used to better identify small defects, which can be technically challenging without visualization aids. After clamping the pulmonary artery, the left ventricle is filled with 100 mL of cold saline mixed with two to three drops of fluorescein dye, allowing the dye to flow out of any defects that were previously unidentified [11]. Fluorescein specifically has improved visualization compared to methylene blue in these cases because methylene blue stains tissue, has a vasoconstrictor effect, and binds to heparin [11]. Further, if the cuff is violated, methylene blue causes persistent tissue staining that lasts several days, potentially compromising visualization during the immediate surgery and creating challenges for subsequent providers visualizing the operative field, especially if they were not involved in the original surgery. To address this visualization issue, Bryant Medical’s TENAX® laser resistant endotracheal tube (ETT) features a cuff pre-filled with blue dye. Fluorescein strips also offer an alternative solution to these same issues while also providing cost-saving advantages.

Fluorescein dye also demonstrates maintained vibrancy and stability, making it a reliable alternative to methylene blue. Fluorescein maintains peak absorption in various solutions, whereas other coloring agents, such as marking pens, fade over time [6]. Notably, fluorescein-dyed solutions retained their color stability over a two-hour period without a downward trend, suggesting that color decay is unlikely over extended durations [6]. This durability is particularly advantageous in the operating room, where consistent visibility is critical. Additionally, fluorescein-stained solutions remain identifiable even under dim light conditions, further ensuring reliability in real-world surgical settings [6].

While ideally the endotracheal cuff will not be violated and the dye will not come into contact with airway mucosa, it is important to consider the safety profile of the dyes in case of cuff perforation. If damage to the endotracheal cuff were to occur, both methylene blue and fluorescein are generally safe on mucosal surfaces [4-6]. Oral administration of fluorescein solution is used in a variety of ophthalmic procedures with a low rate of mild side effects [5]. Side effects include minimal itching, discomfort, and nausea, and all side effects resolve after a 1-hour rest period [5]. When fluorescein is used in the eye, it drains through the nasolacrimal duct into the nasal cavity without adverse effects [6]. It has also been used to visualize cerebrospinal fluid leakage when administered intrathecally as a safe and accurate tool [12,13].

Given the high frequency of laser laryngeal procedures and their broad clinical indications [13], even modest cost savings per case can translate into substantial overall financial benefits. Conservatively estimating that a laryngologist performs 50 laser surgeries requiring laser cuff inflation per year, this would yield a savings of $7,492 after only one year of applying this change. This change is easy to implement and will result in immediate and long-term cost savings for both hospitals and patients.

## Conclusions

By using fluorescein strips, laser laryngeal surgeries can realize significant cost savings in the balloon cuff component of the procedure. We propose using fluorescein dye in place of methylene blue for endotracheal cuff inflation in laser laryngeal surgeries. When used for cuff inflation, fluorescein behaves in the same manner as methylene blue, as a compromised cuff will produce a sudden flash of dyed solution into view and warn the surgeon of a damaged cuff. This change is easy to implement and will result in immediate, long-term cost savings for both patients and hospitals.

Both dyes are safe to use and function effectively to monitor endotracheal cuff integrity. Given their similar mechanisms and safety profiles, fluorescein strips are much less expensive than a vial of methylene blue. Therefore, fluorescein strips can serve as an alternative to methylene blue without compromising function. While direct comparative studies between methylene blue and fluorescein for endotracheal cuff leak detection are limited, the established use of both dyes in similar diagnostic applications supports their interchangeable use in this context. This substitution has the potential to greatly reduce costs while maintaining the same level of clinical reliability in detecting endotracheal cuff leaks.
